# Supplementary material for: The Bulb, the Brain and the Being: New Insights into Olfactory System Anatomy, Organization and Connectivity
Source: Brain Sci. 2025 Mar 31;15(4):368. doi: 10.3390/brainsci15040368 (PMC12025486; doi:10.3390/brainsci15040368)
Supplement: Supplementary file 1 [file brainsci-15-00368-s001.zip › significant correlations cohort 2.pdf]

Pathway	Test	R-value	P-value	Benjamini Hochberg FDR
**IFOFRad**	CG.ORR.AA	0,454725851546719	4,15947421618781×10^−05	0,00457542163780659
**IFOFRmd**	CG.ORR.AA	0,428876244665718	0,000142948060207443	0,00786214331140938
**IFOFRrd**	CG.ORR.AA	0,393798008534851	0,000521954774412986	0,0191383417284762

Table III. Significant correlations between tract metrics (AD, MD and RD) of the right IFOF, and results the oral reading recognition test.
